# Tax alters telomerase activity through both quantitative and qualitative hTERT transcriptional dysregulations

**DOI:** 10.1186/1742-4690-8-S1-A131

**Published:** 2011-06-06

**Authors:** Maroun Karam, Morgan Thenoz, Linda Zane, Céline Vernin, Agnes Lançon, Christiane Pinatel, Eric Wattel, Franck Mortreux

**Affiliations:** 1 Oncovirologie et Biotherapies, UMR5239 CNRS/ENS Lyon/UCBL/HCL, Hopital Pierre Benite, Lyon, France; 2 Laboratory of Molecular Microbiology, National Institute of Allergy and Infectious Diseases, the National Institutes of Health, Bethesda, Maryland 20892, USA; 3 Oncovirologie et Biotherapies, Centre Léon Bérard, Lyon, France

## 

In untransformed cells impaired for DNA damage repair such as Tax positive cells, insufficient telomerase activity (TA) triggers tumorigenic telomere dysfunctions. Here we show that in quiescent HTLV-1+ T cells deriving from carriers, hTERT expression paralleled tax expression, suggesting that Tax promotes an immortalized phenotype in quiescent T cells. Interestingly, PHA-stimulation significantly augmented tax expression in CD4+ (2.6 times, p= 0.022) but not in CD8+ T cells (1.3, ns). 48 hours after PHA stimulation, uninfected and HTLV-1+CD8+ cells augmented their TA and hTERT expression (p<0.03 for each). In contrast PHA possessed no significant effect on TA and hTERT in HTLV-1+CD4+ cells. The PHA-induced fold changes in TA and hTERT expression were respectively 22 and 6.9-folds lower in infected than in uninfected CD4+ T cells, indicating that PHA decreased the level of TA more than 3 folds that of hTERT expression in the sole HTLV-1+CD4+ T cells subset. This hiatus between hTERT expression and TA was reproduced in vitro upon ectopic Tax expression in epithelial cells. We thus supposed that upon PHA-dependent Tax expression, a negative post-transcriptional element targeted hTERT for decreasing TA in HTLV-1+CD4+ T cells. Accordingly we next demonstrated that, beside its repressive effect on the hTERT promoter, ectopic Tax expression strongly redistributed hTERT RNA isoforms towards a significantly increased proportion in inactive isoforms and a significantly decreased proportion in the active A+B+ isoform. Thus via Tax expression, naturally HTLV-1 infected CD4+ T cells undergo both transcriptional and post-transcriptional hTERT modifications that significantly decrease TA in vivo.

